# Design and assessment of lipase-CuO nanoparticle conjugates for enhanced antimicrobial efficacy against clinical pathogens

**DOI:** 10.1186/s12896-025-00950-0

**Published:** 2025-02-07

**Authors:** Eman M. Handak, Dina H. Amin, Mai M. Elhateir

**Affiliations:** 1 Botany and Microbiology Department, Faculty of Science, Al-Azhar University (Girls Branch), Cairo, 11754 Egypt; 2 Microbiology Department, Faculty of Science, Ain Shams University, Cairo, Egypt

**Keywords:** Antimicrobial agents, Clinical pathogens, Lipase-CuO nanoparticles, Statistical optimization

## Abstract

In the battle against clinical infections particularly the resistant pathogens, the creation of new antimicrobial drugs is essential. This study focuses on synthesis and characterization of Lipase-CuO nanoparticle conjugates in order to investigate their antibacterial efficiency. Lipase enzyme and CuO nanoparticles were synthesized biologically by specific selected fungal strains. Statistical optimization of lipase enzyme was done using a Plackett-Burman design giving two enhancement models for lipase production with increasing in productivity up to 143.43% (2800 U/ml). Copper oxide (CuO) nanoparticles were characterized using visual indication of greenish color formation, UV-vis spectrum analysis which revealed a strong peak at 300 nm. Also, CuO nanoparticles appeared as distinct, well-dispersed spherical particles with average size of 71.035 nm using TEM, while conjugate appears as large protein molecules linked to the nanoparticles. Also, using techniques like energy dispersive X-ray (EDAX) the resultant conjugates formation was confirmed as the elemental analysis approved its formation. The antimicrobial activity of Lipase-CuO nanoparticles conjugates was tested against a range of clinical pathogens. The results demonstrated a significant increase in antimicrobial potency compared to both CuO nanoparticles and lipase alone particularly against *E. coli* strain NRC B-3703 with remarkable increase of 373.6% and 75% followed by *S. aureus* with increase of 50 and 42.8%compared to that of individual CuO nanoparticles and lipase enzyme, respectively. These findings suggest that Lipase-CuO nanoparticle conjugates hold great promise as a novel antimicrobial strategy, offering a potential solution to combat bacterial infections, especially those caused by multidrug-resistant strains. The study highlights the importance of nanotechnology in enhancing the efficacy of traditional antimicrobial agents and opens new avenues for targeted antimicrobial therapies.

**Supplementary Information:**

The online version contains supplementary material available at 10.1186/s12896-025-00950-0.

## Introduction

Microbial lipases are versatile enzymes with a wide range of industrial applications. Through processes like interesterification, esterification, aminolysis, and alcoholysis, they can break down lipids into smaller components like fatty acids and glycerol, producing new molecules that have beneficial effects across different sectors including food, detergents, pharmaceuticals, leather, textiles, cosmetics, biodiesel production, and paper manufacturing [1]. Lipases are highly efficient catalysts capable of functioning in both aqueous and non-aqueous media. Their exceptional stability across a wide range of temperatures, pH values, and organic solvents and their remarkable selectivity for specific chemical reactions without requiring additional cofactors enhances their versatility. The hydrophobic lid structure of lipases is essential for their interfacial activity. These enzymes are classified according to their specificity and source, with various plants, animals, insects, and microorganisms producing them [2–7].

Several researches have been reported lipase production by various types of microorganisms including bacteria (e.g., *Bacillus*,* Pseudomonas*,* Burkholderia*) [8–11], fungi (e.g., *Penicillium*,* Fusarium*,* Aspergillus*) [12–15], and yeast [16–18]. Bacteria commonly secrete lipases into the culture medium through submerged fermentation, while fungal lipases are often extracellular or intracellular and require more complex extraction processes [19]. Yeast-derived lipases can be intracellular, extracellular, or membrane-bound, and are often valued for their stability and specificity [20]. Microbial lipases are favored in industry due to their diverse catalytic activities, high production yields, amenability to genetic engineering, and robust stability [21]. In this study, we applied a factorial design approach to optimize lipase production using limited resources.

Factorial designs are commonly employed by researchers to examine the effects of multiple factors, such as temperature, pH, and inducer concentration, on lipase production as each isolate respond differently to the growing physical and nutritional factors, highlighting the importance of the optimization process using statistical methodologies saving time, effort and cost [22, 23]. Response Surface Methodology (RSM) further optimizes process parameters by fitting mathematical models to experimental data, predicting optimal conditions for maximum lipase yield, as seen in studies on *Bacillus brevis* lipase production [24]. Additionally, the Plackett-Burman design is used as a screening method to identify key factors influencing lipase production, such as pH, inoculum size, and incubation time, in *Pichia guilliermondii*. Mathematical models developed from these techniques help predict lipase production and are invaluable for process improvement and control [25].

The escalating prevalence of antibiotic-resistant bacteria presents a critical global health challenge, demanding the development of alternative antimicrobial strategies. Metal oxide nanoparticles, especially copper oxide (CuO), have emerged as promising candidates due to their potent antimicrobial efficacy against various pathogens, particularly effective against drug-resistant strains, making them valuable in combating antibiotic-resistant infections [26–28].

In this manuscript, the importance of using factorial designs for lipase production by *Penicillium sp* was highlighted as it was selected from previous studies as an efficient strain that can grow on oil -based medium with high potential growth; the optimal levels for each factor can be identified to achieve maximum lipase production, which is crucial for industrial applications. Furthermore, Factorial designs offer an efficient and economical approach to exploring multiple variables simultaneously, conserving time and resources [23]. This method effectively quantifies the impact of individual factors and their interactions, providing invaluable data for process optimization [29].

This study aims to investigate the antimicrobial efficacy of novel conjugates composed of lipase and CuO NPs. The conjugation of lipase with CuO NPs can enhance the synergistic effects of both components, particularly their antimicrobial activity compared to using each product individually which provide insights into its potential applications in combating antibiotic-resistant pathogens and to be used as a potent antimicrobial agent. Also, this is a chance to get rid of waste oil sources which are cheap as castor or causing deleterious effects on the environment as frying and engine, along with the formation of valuable products using microorganisms as a benign green method.

## Materials and methods

### Evaluation of *Penicillium griseofulvum* P-1707’s growth potential on various inexpensive oil sources

The growth ability of *Penicillium griseofulvum* P-1707 on various cheap oil sources was evaluated using a specifically formulated production medium. The medium composition per liter included 1 g KH₂PO₄, 1 g MgSO₄0.7 H₂O, 35 g NH₄Cl, 5 g yeast extract, and 20 g agar. Additionally, 1% (v/v) of different waste oil sources (frying oil, castor oil, and engine oil) were separately incorporated into the medium, maintaining a pH of 6. The prepared media were sterilized at 121 °C under 1.5 atm pressures for 20 min. After sterilization, the media were cooled to 45 °C and poured into sterilized petri dishes. Inoculation involved streaking the surface of the plates with *Penicillium griseofulvum* P-1707. The inoculated plates were incubated at 28 °C for a period of 7 days, as described by [30]. The strain *Penicillium griseofulvum* P-1707 used in this study was obtained from previous research and had been genetically identified [31].

### Production and extraction of lipase enzyme

The production medium contained the same components as those used for screening growth; with the exception that agar was omitted. Broth medium was prepared and distributed into flasks, each containing 100 ml medium and 1% of oil sources (frying oil, castor oil, or engine oil) was added, two flasks for each oil source, and one flask as a control. All flasks were sterilized by autoclaving.

Following sterilization, each flask was inoculated with 2 loopfuls of the fungal strain *Penicillium griseofulvum* P-1707 and incubated at 28 °C for 7 days. After the incubation period, the contents of the flasks were filtered using Whatman filter paper no. 1. The mycelium was dried overnight at 60 °C to determine the mycelia dry weight, which served as a measure of fungal growth on each waste oil source. The cell-free filtrate was stored in sterilized Falcon tubes at 0 °C for subsequent determination of lipase productivity.

### Lipase enzyme assay using chromogenic substrate plates and a titrimetric method

#### Chromogenic substrate plates

Chromogenic substrate plates were prepared using phenol red (0.01%), 1% oil substrate (tributyrin), 10 mM CaCl_2_, and 2% agar. The pH was adjusted to 7.3–7.4 with 0.1 N NaOH. A 0.1 ml sample of the crude enzyme from each flask was added to the central well of the plate. The plates were then incubated at 37–45 °C for 30 min. After incubation the plates which give yellow zone indicates lipase production (as change of phenol red color as a result of acid production) [32].

#### Titrimetric method

The titrimetric method contained olive oil as the substrate and thymolphthalein as the indicator. The endpoint was determined by the color change of the reaction mixture from colorless to pale blue. To prepare the substrate emulsion, 25 ml of olive oil and 75 ml of 7% Arabic gum solution were emulsified in a blender for 2 min. The reaction mixture contained 5 ml of the olive oil emulsion, 2 ml of 0.1 M phosphate buffer (pH 7.0), and 1 ml of the enzyme suspension, and was incubated at 37 °C for 30 min with orbital shaking. Immediately after incubation, the emulsion was disrupted by adding 15 ml of acetone-ethanol (1:1 v/v), and the liberated free fatty acids were titrated with 0.1 M NaOH. One unit of enzyme activity (U) was defined as the micromoles of free fatty acids released under the assay conditions and expressed as U/ml per hour of incubation [30].

The enzyme activity (U/ml) was calculated using the formula:

$$\eqalign{{\rm{U/ml}}\,{\rm{of}}\,{\rm{enzyme}}\,{\rm{activity}}\,{\rm{ = }} & {\rm{ (NaOH}}\,{\rm{volume }} \cr & \times \,{\rm{molarity}}\,{\rm{of}}\,{\rm{NaOH}} \cr & \times \,{\rm{1000}}\, \times \,{\rm{2)}} \cr} $$

Where: 1000 is the conversion factor from milli-equivalents to micro-equivalents, 2 is the conversion factor from 30 min to 1 h.

### Statistical optimization of lipase production by *Penicillium griseofulvum* P-1707 using Plackett-Burman design

The statistical optimization of lipase was conducted using a Plackett-Burman design model [33] to evaluate the effects of five factors on lipase production including one oil source (Castor) using Design- Expert software (version 13, Stat-Ease, Inc., Minneapolis, MN) as indicated in Table S1. The factors and their levels were: Factor 1 (A: Temperature): 25 °C, 50°; Factor 2 (B: Initial pH): 4, 8; Factor 3 (C: Incubation Time, Days): 2, 8; Factor 4 (D: Inoculum Size, %): 1, 10 and Factor 5 (E: Castor Oil, %): 1, 5.

Another trial for achieving highly efficient lipase statistical production model was a second design including the three experimented oil sources (Castor, Frying and Engine) was performed. Seven factors viz. (incubation temperature; initial pH, incubation time, frying oil, Engine oil and castor oil concentrations) were primarily introduced into plackett-Burman design using Design- Expert software (version 13, Stat-Ease, Inc., Minneapolis, MN). Fifteen runs were done according to measuring lipase productivity for each using titrimetric method (Table S2). The obtained results were analyzed using ANOVA analysis. The significant factors were identified. The design was validated [33].

**The second design**: The second design model assessed the effects of seven factors on lipase production: Factor 1 (A: Temperature): 20 °C, 50 °C; Factor 2 (B: Initial pH): 4, 8; Factor 3 (C: Incubation Time, Days): 2, 8; Factor 4 (D: Inoculum Size, %): 1, 10; Factor 5 (E: Frying Oil, %): 1, 5; Factor 6 (F: Engine Waste Oil, %): 1, 5 and Factor 7 (G: Castor Oil, %): 1, 5.

### Purification of lipase

Cell free filtrate was prepared followed by ammonium sulphate precipitation at 80% concentration. The precipitated protein was undergoing dialysis against 0.1 M phosphate buffer pH7 with continuous buffer renewing every 2–3 h for 24 h then, the crude lipase was concentrated against sugar crystals. The concentrate was re-suspended in minimal volume of the 0.1 M phosphate buffer pH7 to be applied on the sephadex G-200 column of 2.2 × 63 cm. lipase activity and protein concentration was measured for all fractions [30].

### Production of copper oxide nanoparticles

#### Isolation of copper oxide nanoparticles fungal producers

Five isolates were obtained from a soil sample collected from a detergent industry site at Savo Factory, Al Amereia Area; Cairo, Egypt. The isolates were tested for CuO nanoparticle production, the isolate which exhibited the highest growth on malt extract agar medium when incubated at 30 °C for 7 days was selected. The isolate was subsequently purified and cultivated under the same medium and conditions for further study [34].

#### Genetic identification of the selected copper oxide nanoparticles producing isolate

The isolate was cultured in 35 mL of malt extract broth and incubated for 7 days at 30 °C in a shaking incubator. DNA extraction was conducted using DNeasy kit (Qiagen). DNA was stored at -20 °C for further manipulation. Taq PCR Master mix (purchased from Qiagene) was used to amplify or synthesize DNA (genomic or plasmid) fragments using a thermal cycler machine (gradient Robocycler 96 Stratagene, USA) at the Regional Center for Mycology and Biotechnology.

The interested DNA fragments (PCR products and linear plasmid) were purified by agarose gel electrophoresis using the appropriate gel concentration stained with ethidium bromide. The gel was run to the desired level of voltage and the DNA was visualized and imaged using the transilluminator of a gel documentation system (BIO-RAD, Gel Doc 2000).

Sequencing of plasmid and amplified PCR fragments was carried out by Cy5/Cy5.5 Dye Primer Sequencing kit from Visible Genetics Inc. for use with the Open Gene automated DNA sequencing system [35].

Filling, casting, polymerizing Micro Cell Cassette, loading the Micro Cell Cassette into Long-Read Tower, and electrophoresis conditions were assigned according to Long-Read Tower DNA sequencer System and sample analyses were made by OpenGene software Version 3.1 from Visible Genetics, Canada at The Regional Center for Mycology and Biotechnology.

Primer sequences used for the identification of 18s in the current study.

ITS1( 5’- TCC GTA GGT GAA CCT GCG G-‘3)

ITS4 (5’- TCC TCC GCT TAT TGA TAT GC-‘3)

### Production, extraction and characterization of copper oxide nanoparticles

C’zapeck Dox medium was prepared in a 250 ml conical flask, sterilized, inoculated with 5% inoculum, and incubated at 30 °C for 7 days. The culture was then filtered using Whatman filter paper no.1. Under aseptic conditions, 10 ml of 50 mM copper sulfate was added to the cell-free filtrate and incubated for 3 days under the same conditions. The strain used for production was previously isolated and genetically identified based on 18s rRNA. The cell-free filtrate was centrifuged at 10,000 rpm for 10 min at 4 °C. The resulting pellet was washed with isopropanol and centrifuged again under the same conditions. A dark brownish-black precipitate of CuO nanoparticles was obtained, dried at 50 °C overnight, and subjected to characterization [36]. The morphology and dimensions of the CuO nanoparticles were analyzed using visual observation of color changing, UV-Vis absorption spectroscopy (Genway 6300, UK) across a range of 200–700 nm, Transmission electron microscopy (TEM), Energy Dispersive X-ray (EDAX) and Fourier-transform infrared spectroscopy (FTIR) to confirm their morphology, crystallinity, and functional groups.

### Preparation and examination of lipase-CuO mixture

5 ml mixture was prepared by suspending 3.2 mg of CuO nanoparticles in 5% DMSO at 4 °C. Then, 1 ml of purified lipase enzyme (concentration 1.4 µg/ml) was added, and the mixture was maintained at the same temperature for analysis. The lipase-CuO mixture was examined using EDAX and TEM (JEOL JEM 2100, 200 keV – 0.143 resolution TEM).

### Antimicrobial activity of lipase-CuO nanoparticles Conjugate

The antimicrobial effect of the lipase-CuO nanoparticles conjugate was tested against five pathogenic bacterial strains Two Gram-positive bacterial strains (*Staphylococcus aureus* NRRL B-313 and *Bacillus subtilis* NRC), and three Gram-negative strains (*Escherichia coli* NRC B-3703, *Salmonella typhimurium* ATCC 14028 and *Pseudomonas aeruginosa* NRC B-32)., three fungal strains (*Fusarium chlamydosporum* F25, *Aspergillus terrus* SQU14026 and *Alternaria alternata* Te19)., and one yeast strain *Candida albicans* NRRL477 using the agar diffusion well method [37]. Seeded medium was used for bacterial strains and yeast, while fungal strains were streaked on the surface of the plates. Three experimental groups were established in addition to the control: lipase, CuO nanoparticles and the lipase-CuO nanoparticles mixture. Each sample (0.1 ml) was applied to the wells, and the plates were incubated at 30 °C for 2 days for bacteria and yeast, and 7 days for fungal strains. Two control sets were provided; the first set was the inoculated plates without addition of the tested samples; and with the addition of 0.1 ml DMSO 5% in order to ensure that the resulted effect is due to the action of the tested samples only as a negative control. The second group was with the addition of Ampicillin and Mycostatin as positive controls for both bacterial and fungal strains with concentrations of 50 and 100 µg/ml, respectively (Table S5). Also, the test was carried out using the crude lipase (1.7 mg/ml) for getting adequate concentration; the lipase-CuO nanoparticles mixture was prepared with the same method as mentioned in the previous step.

### Minimum inhibitory concentrations (MIC) and minimum bacterial concentrations (MBC)

The antimicrobial activity of the three tested substances (lipase only, CuO-NPs only and lipase CuO-NPs -conjugate) was determined by using the standard broth dilution method (CLSI M07-A8). BHI broth was used for MIC calculation by twofold serial dilutions of the tree tested substances. Different concentrations of were used starting lipase from (1700 µg/mL to 53.13 µg/mL), CuO-NPs from(1600 µg/mL to 50 µg/mL) with an adjusted bacterial concentration 0.5 McFarland’s standard of (1 × 108 CFU/ml) at 625 nm Tested bacterial growth in BHI broth was used as positive control and only broth media was used as a negative control. The MIC was determined visually by the turbidity of the tubes before and after the incubation for 24 h for bacterial growth and 48–72 h for fungal growth. After the MIC measurement of the three tested substances (lipase only, CuO-NPs only and lipase CuO-NPs -conjugate), 50 µl sample from all the tubes was swabbed on plates; the plates that showed no visible bacterial growth were considered to be MBC.

## Results

### The effect of different oil sources on microbial activity and lipase production

**Fig. 1:**
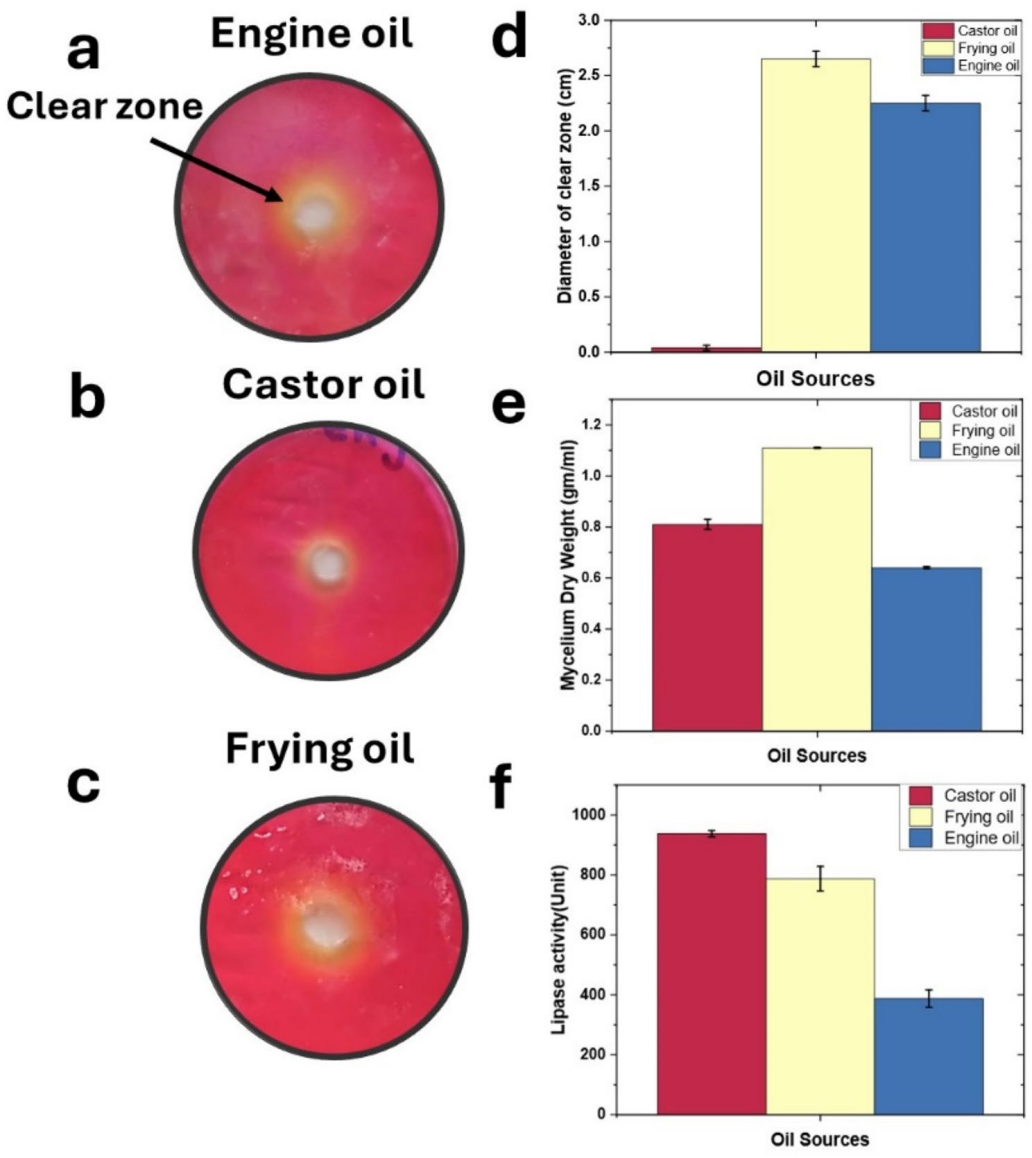
Effect of different oil sources on microbial activity and lipase production. Panels (**a-c**) show the clear zones indicating lipid degradation on agar plates inoculated with *Penicillium griseofulvum* P-1707 and supplemented with (**a**) Engine oil, (**b**) Castor oil, and (**c**) Frying oil. Panel (**d**) quantifies the diameter of the clear zones for each oil source, Panel (**e**) presents the mycelium dry weight for each oil source, Panel (**f**) shows the lipase activity (U/mL) for each oil source. Error bars represent standard deviations from the means of triplicate experiments

Clear Yellow zone formation on agar plates supplemented with castor oil, engine oil, and frying oil indicated varying degrees of lipid degradation. Frying oil and engine oil showed a pronounced clear zone (3.15 cm) followed by frying (2.65 cm) and engine (2.25 cm) (Fig. 1a, b, c). Frying oil yielded the highest mycelium production at 1.11 g/100 ml, followed by castor oil at 0.81 g/100 ml, and engine oil at 0.64 g/100 ml. In terms of lipase productivity using titrimetric assay, castor oil exhibited the highest activity with 937.6 U/ml, followed by frying oil with 787.6 U/ml, and engine oil with 387.6 U/ml (Fig. 1d-f). These results indicate that castor oil is the most effective substrate for promoting microbial lipid degradation and lipase production, followed by frying oil, while engine oil is the least effective. Also, the most suitable substrate for optimum growth for *Penicillium griseofulvum* P-1707 was frying oil followed by castor and then, engine.

### Statistical optimization of lipase production by *Penicillium Griseofulvum* P-1707 using Plackett-Burman design

The Titrimetric assay method was used for measuring lipase production by *Penicillium griseofulvum* P-1707 for all 15 runs that obtained from a factorial design model (Plackett-Burman) to evaluate the effects of five factors: temperature, initial pH, incubation time, inoculum size, and castor oil concentration using Design- Expert software (version 13, Stat-Ease, Inc., Minneapolis, MN).

### ANOVA for first design model

**Fig. 2:**
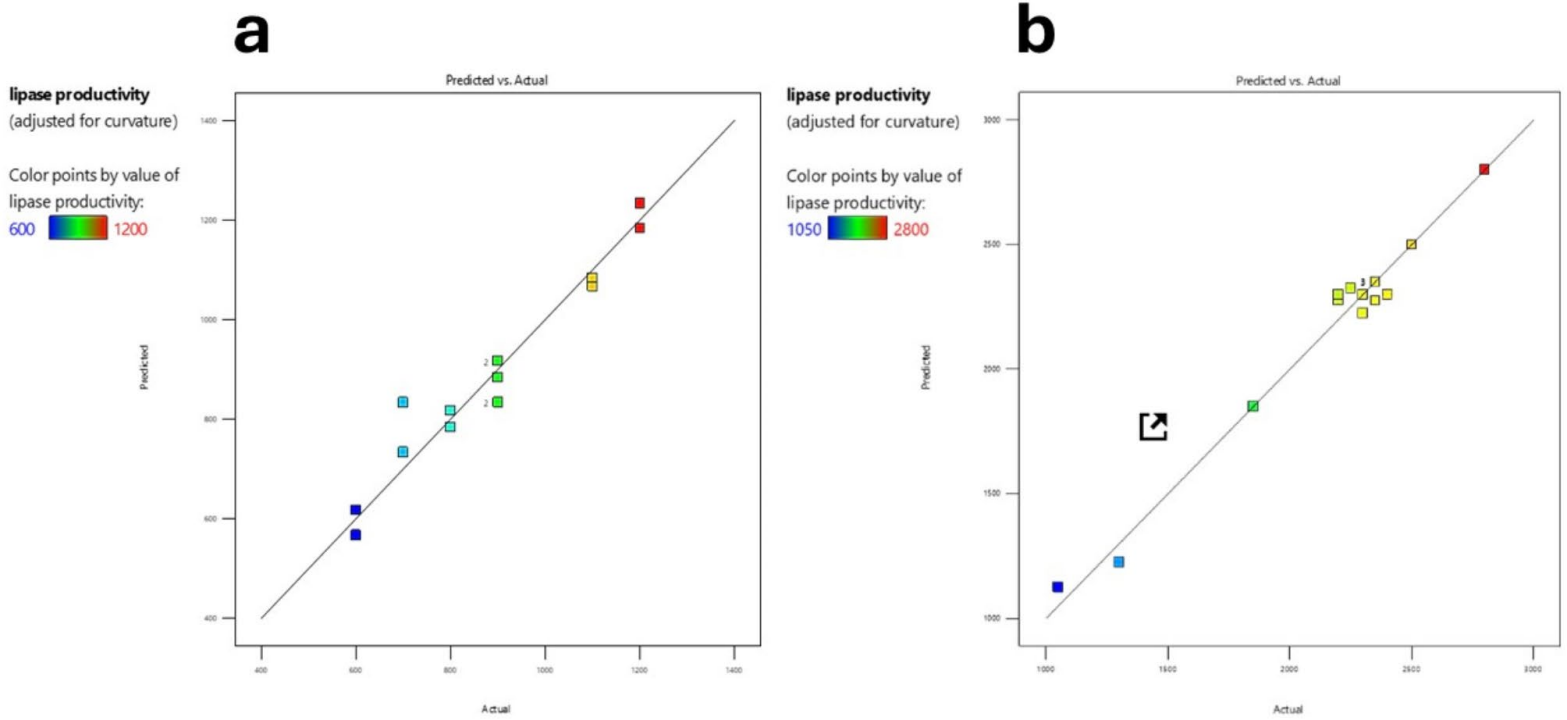
Correlation between Predicted and Actual Lipase Productivity. (**a**) Plot of predicted versus actual lipase productivity values, with a color gradient representing productivity levels ranging from 600 to 1200U/ml. The data points are closely clustered around the diagonal line (y = x), indicating a strong linear relationship and high predictive accuracy of the model. (**b**) Similar plot with productivity values ranging from 1050 to 2800U/ml, demonstrating the model’s consistent performance across a different range of productivity values. The color gradient from blue to red effectively highlights the variations in lipase productivity

The ANOVA results for the first design model, which evaluated lipase productivity, are presented in (Table S3). The model was significant with a p-value of 0.0427, indicating a good fit. The model’s F-value of 6.58 indicates its significance. Significant model terms included temperature (A), castor oil (E), and interaction terms BE and CE (Fig. 2). A Lack of Fit F-value of 0.25, which is not significant, suggests the model fits well. The temperature (p-value = 0.0159), castor oil (p-value = 0.0341), and interaction terms pH * Castor oil (BE) with p-value = 0.0090, and Incubation time * Castor oil (CE) with p-value = 0.0159 were significant factors affecting lipase production. An adequate Precision ratio of 8.528, which is greater than the desirable ratio of 4, confirms an adequate signal, Also, the model R² = 0.94 The model and adjusted R^2^ = 0.8 are very close making the model suitable for navigating the design space.

Final Equation in Terms of Actual Factors for the first design:

**lipase productivity (U/ml)** = + 443.05556–0.500000 temperature + 90.62500Initial pH -64.58333 Incubation time + 23.61111 Inoculum size + 340.27778 Castor oil − 3.50000 temperature * Castor oil − 40.62500Initial pH * Castor oil + 22.91667 Incubation time * Castor oil − 6.94444 Inoculum size * Castor oil.

### ANOVA for second design model

**Fig. 3:**
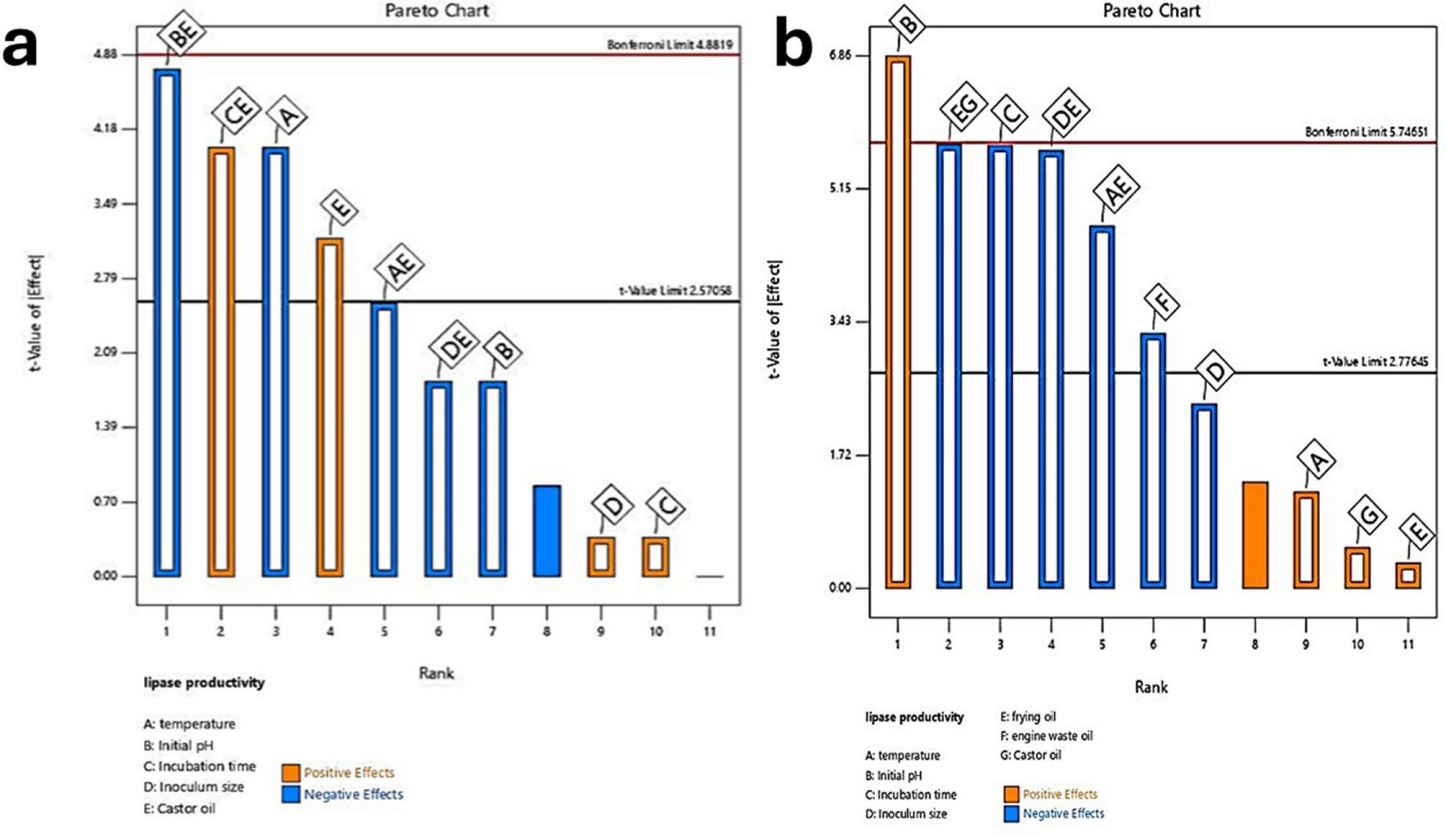
Pareto charts of the Plackett-Burman design illustrating the effects of different factors on lipase enzyme productivity by *Penicillium griseofulvum* P-1707. The chart illustrates the relative significance of each factor, with bars representing the absolute values of their standardized effects. (**a**) Pareto chart from the first Plackett-Burman design, showing the impact of factors A (temperature), B (initial pH), C (incubation time), D (inoculum size), and E (castor oil). Positive effects are shown in orange, and negative effects are shown in blue. The Bonferroni and t-value limits indicate statistical significance, with factors E, A, and B being the most significant contributors to lipase productivity. (**b**) Pareto chart from the second Plackett-Burman design, evaluating factors A (temperature), B (initial pH), C (incubation time), D (inoculum size), E (frying oil), F (engine waste oil), and G (castor oil). The chart shows significant positive and negative effects, with factors B, C, D, and F represented the highest contributors to lipase productivity in the design

The second design model’s ANOVA results (Table S4 and Fig. 3) evaluated additional factors, including frying oil and engine waste oil, showing significant influence on lipase productivity. Herein, the model F-value of 15.22 implies the model is significant, the model p-value = 0.0231. Also, B, C, F, AE, DE, EG were found to be significant model terms indicated by their p-values (less than 0.0500) (B-Initial pH (p-value = 0.0063), C-Incubation time (p-value = 0.0107), F-engine waste oil (p-value = 0.0462), temperature * frying oil (p-value = 0.0186), Inoculum size * frying oil (p-value = 0.0110) and frying oil * Castor oil (p-value = 0.0106); as indicated in (Fig. 3).The Lack of Fit F-value of 3.38 implies the Lack of Fit is not significant. Also, R2 = 0.98 is very close to adjusted R2 (0.92). Herein, the adequate precision ratio of 13.991 indicates an adequate signal. This model can be used to navigate the design space.

Final Equation in Terms of Actual Factors for the second design.

**lipase productivity (U/ml)** = -924.47917 + 36.87500 temperature + 187.50000 Initial pH -137.50000 Incubation time + 129.16667Inoculum size + 980.03472 frying oil − 118.75000 engine waste oil + 320.31250 Castor oil − 11.04167 temperature * frying oil − 51.38889 Inoculum size * frying oil − 101.56250 frying oil * Castor oil.

The figure presents two subplots (2a and 2b) that represent the correlation between predicted and actual lipase productivity values for the designs. Both subplots illustrate a strong linear relationship, indicating the predictive model’s accuracy. The data points closely cluster around the diagonal line (y = x) in both subplots, suggesting that the model’s predictions are consistent with the actual measurements. This consistency demonstrates the model’s reliability in predicting lipase productivity. Overall, the figure highlights the model’s high accuracy and effectiveness in forecasting lipase productivity across different productivity ranges.

### Validation for the first design

The optimization of the first design parameters resulted in a significant increase in lipase productivity. The selected solution with a temperature of 46.9 °C, an initial pH of 4.1, an incubation time of 7.9 days, an inoculum size of 2.6%, and a castor oil concentration of 4.9% predicted a lipase productivity of 1212 U/ml with a desirability of 1.000. The obtained experimental result was 1150 U/ml, achieving a validation percentage of 95%. This optimization led to a 22.7% increase in lipase productivity from 937.6 to 1150 U/ml.

### Validation for the second design

For the second design, the optimization process also resulted in a substantial further increase (143.43% more than first design result) in lipase productivity. The obtained experimental result was 2800 U/ml, while the predicted productivity was 3212 U/ml with a validation percentage of 87.17%. The optimum conditions were at a temperature of 50 °C, an initial pH of 8, an incubation time of 5.0 days, an inoculum size of 1%, and concentrations of 1% for the tree oils (frying, engine waste and castor oils).

### Purification pattern of lipase

The purification process of lipase showed the following results: starting with a crude extract having a total activity of 700,000 units, total protein content of 444.68 mg, and a specific activity of 1574.18 units/mg, resulting in 100% recovery. After ammonium sulfate ((NH₄)₂SO₄) precipitation, the total activity was reduced to 450,000 units, with a total protein content of 201.9 mg and a specific activity of 2,228.8 units/mg, achieving a 1.42-fold purification and 64.29% recovery. Further purification using Sephadex G-200 resulted in a total activity of 55,000 units, a total protein content of 0.36 mg, and a specific activity of 152,778 units/mg, leading to a 97.05-fold purification and 7.86% recovery.

**Fig. 4:**
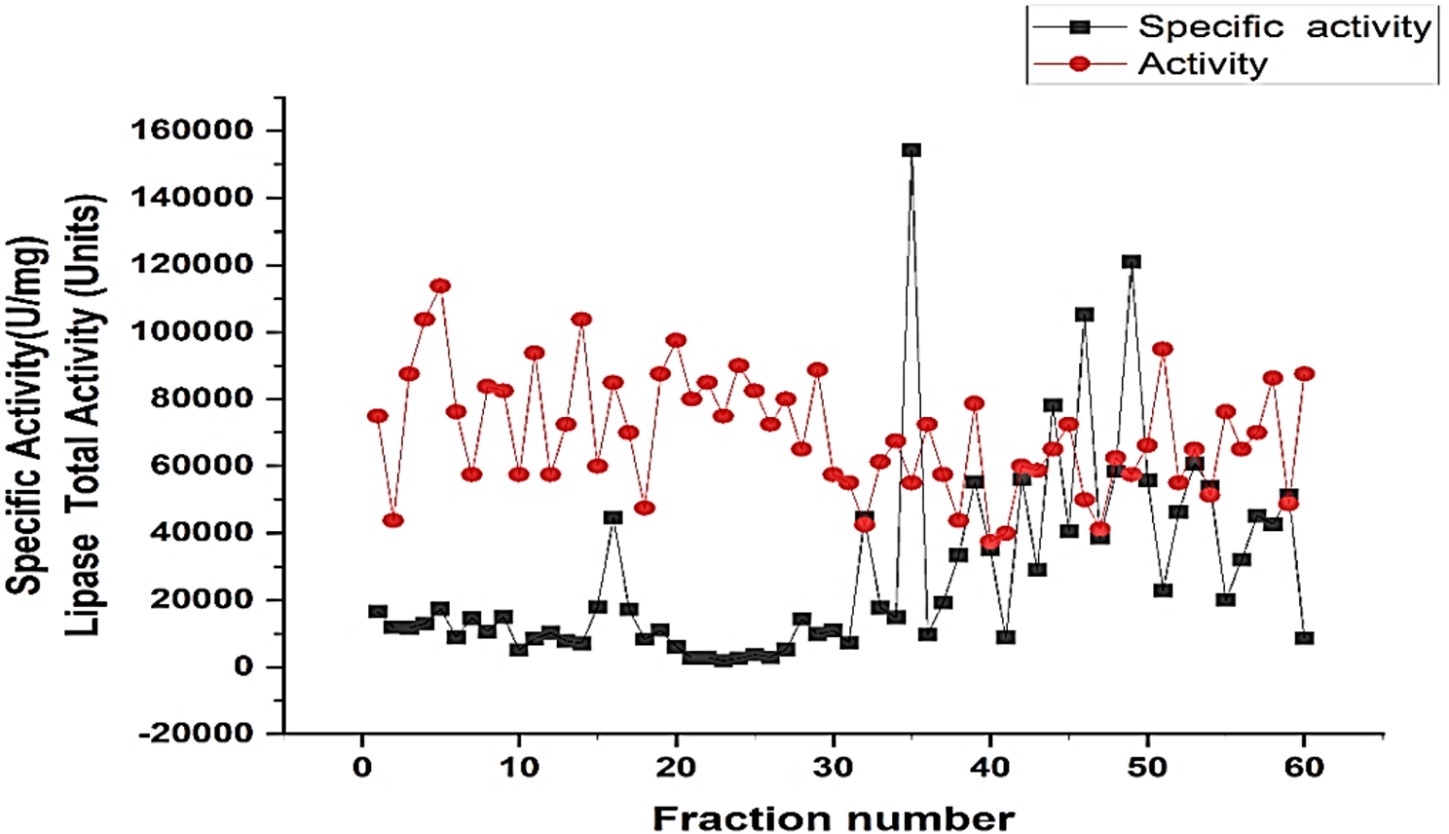
Lipase activity and specific activity across fractions. The fractionation results display the lipase total activity (units) and specific activity (U/mg) across 65 fractions. The data shows fluctuations in both activities, with peaks indicating the fractions with the highest enzymatic activity. These results validate the efficacy of the purification process, highlighting the fractions that exhibit the highest lipase activity and specific activity

The specific activity and total activity were tracked across 65 fractions. The data shows fluctuations in both activities, with peaks indicating the fractions with the highest enzymatic activity. These results validate the efficacy of the purification process, highlighting the fractions that exhibit the highest lipase activity and specific activity. The most active fraction was observed at fraction 35, demonstrating the concentration of highly active lipase (Fig. 4).

### Molecular identification and phylogenetic tree of selected copper oxide nanoparticles producing isolate

**Fig. 5:**
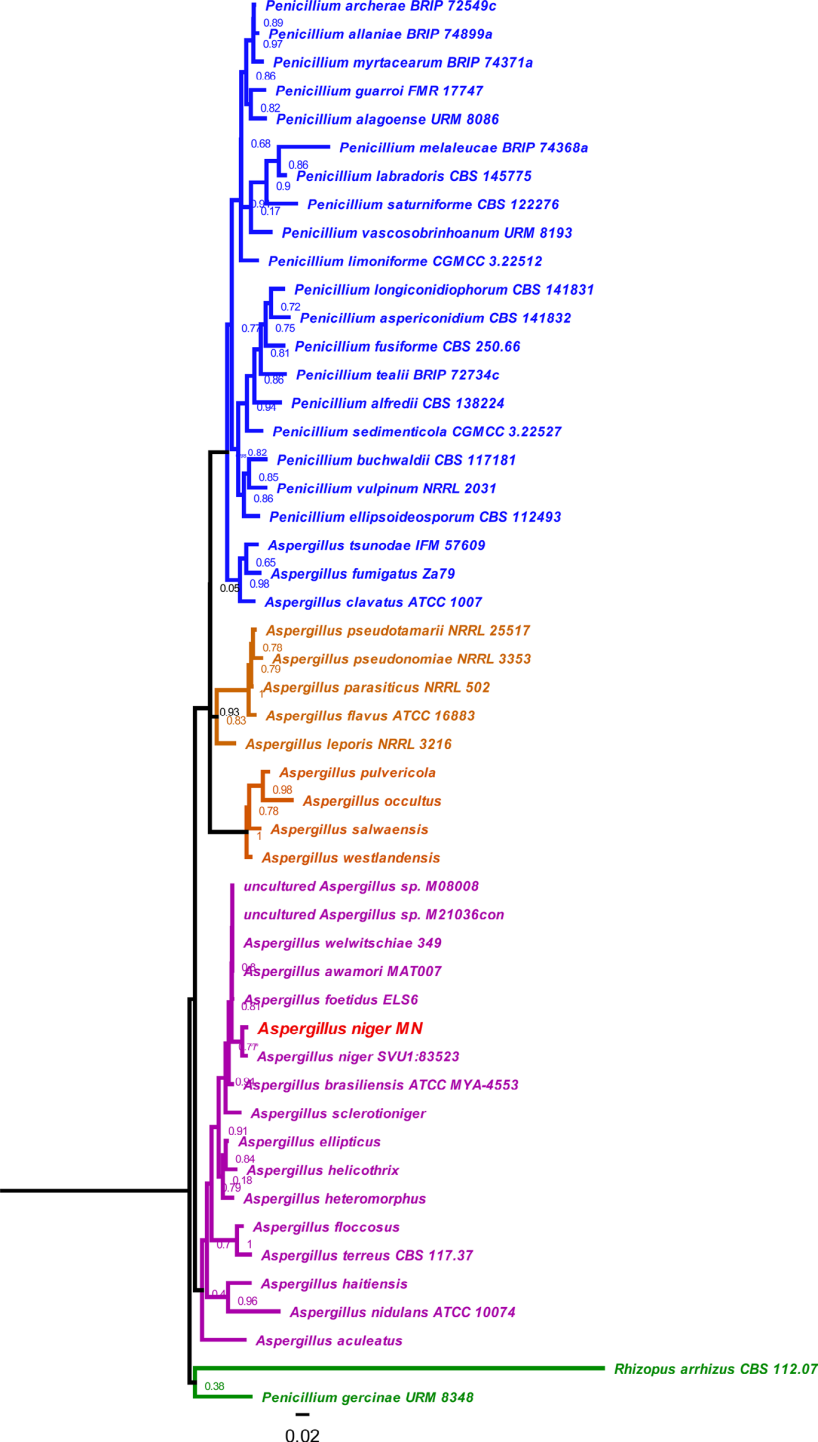
Phylogenetic Tree of *Aspergillus niger* MN. The phylogenetic tree is based on the partial sequence of the small subunit ribosomal RNA gene, complete sequence of the internal transcribed spacer 1 and 5.8 S ribosomal RNA gene, and partial sequence of internal transcribed spacer 2. The ITS sequences were deposited in the GenBank database (http://blast.ncbi.nlm.nih.gov/, accessed on 10 July 2024) to identify closely related species. They were used to build a maximum likelihood phylogenetic tree with the default multiple sequence alignment methods, and the tree inference method was FastTree 2.1.1 builder (Price et al., 2009) in Geneious 9.1.8 software (http://www.geneious.com/). Branch lengths are proportional to nucleotide substitutions/site, and trees were visualized in iTOL v6.3 software (https://itol.embl.de/)

The molecular identification of *the* selected isolate was conducted using sequences of the small subunit ribosomal RNA gene (partial sequence), internal transcribed spacer 1 and 5.8 S ribosomal RNA gene (complete sequence), and internal transcribed spacer 2 (partial sequence), deposited under GenBank accession PP792979.1. Phylogenetic analysis placed *A. niger* MN PP792979.1 within the lower clade of the phylogenetic tree. This clade exhibited evolutionary proximity to *A. niger* SVU1:83523 and showed conservation within *Aspergillus* spp., encompassing species such as *A. brasiliensis*,* A. awamori*,* A. foetidus*,* A. sclerotioniger*, and *A. welwitschiae* as shown in (Fig. 5).

Interestingly, *A. niger* MN PP792979.1 has a unique relationship with uncultured *Aspergillus* strains, suggesting specialized genetic and biosynthetic capabilities potentially related to distinctive nutritive traits. This evolutionary context underscores the strain’s potential for novel metabolic pathways and secondary metabolite production, indicative of its adaptation to a specific ecological niche.

**Fig. 6:**
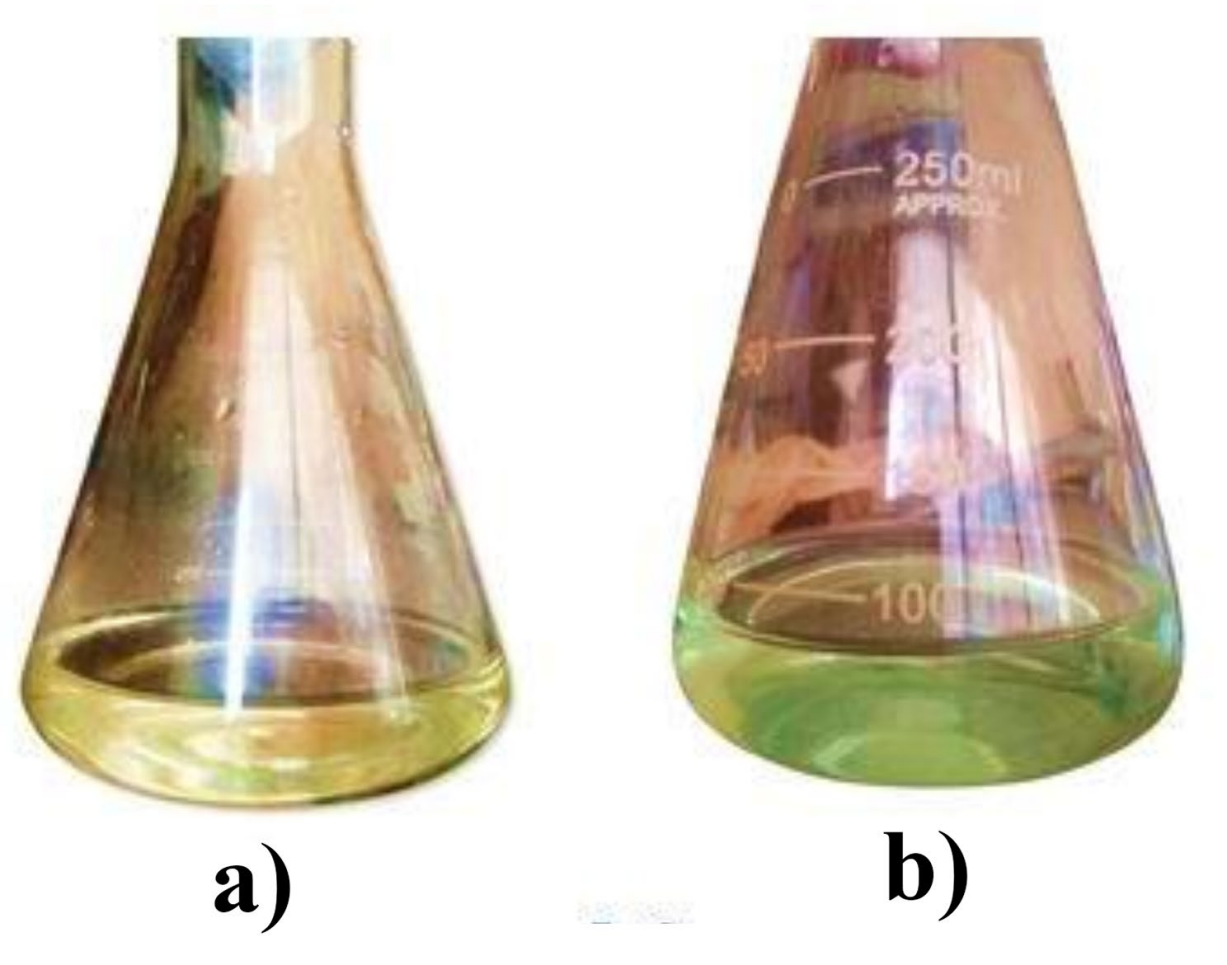
Visual image of (**a**) filtrate of *Aspergillus niger* MN PP792979.1 before CuSO4 addition, (**b**) after addition of CuSO4. Greenish color formation in b is as a result of CuO nanoparticles formation after the addition of 50 mM copper sulfate comparing to the light yellow color of the filtrate

**Fig. 7:**
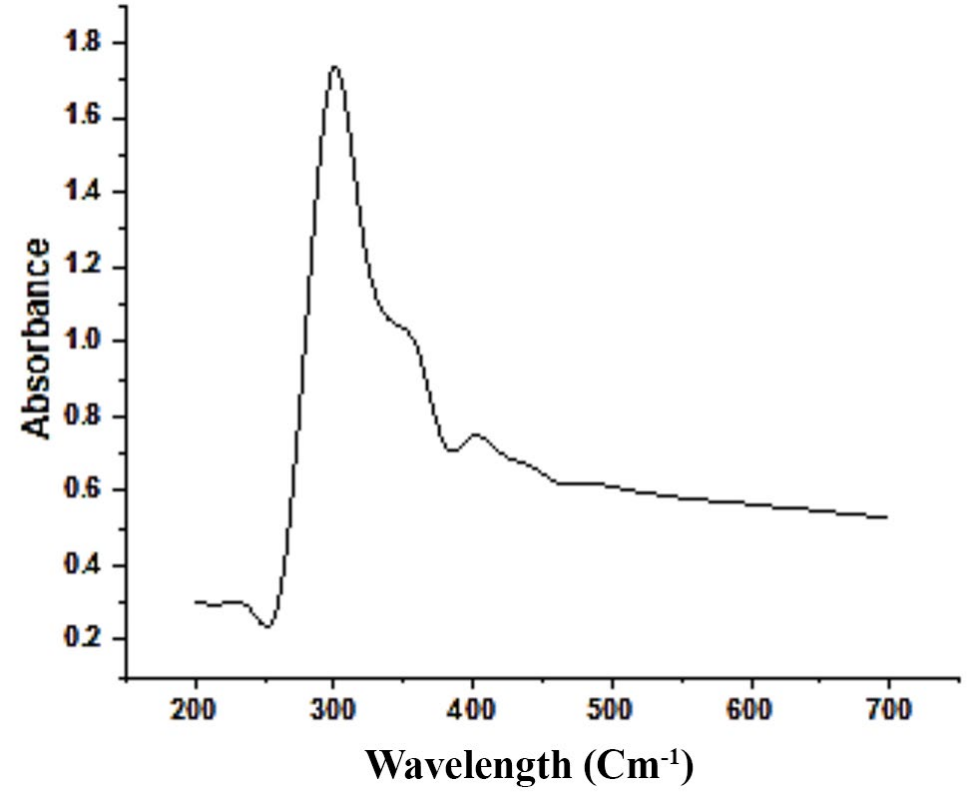
UV-Vis spectrum of CuO nanoparticles produced by *Aspergillus niger* MN PP792979.1. The results revealed a strong peak at 300 nm corresponding to CuO nanoparticles formation at a range characteristic for them

The formation of CuO nanoparticles by *Aspergillus niger* MN PP792979.1 was firstly characterized using visual observation of color change into greenish (Fig. 6). In addition, UV-Vis absorption spectroscopy (Genway 6300, UK) revealed a strong absorbance peak at 300 nm across measured range of 200–700 nm suggesting the formation of CuO NPs (Fig. 7).

### Fourier-transform infrared (FTIR)

**Fig. 8:**
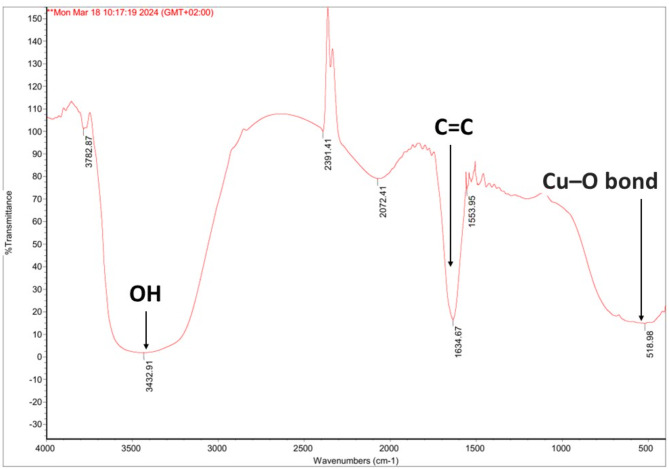
FTIR spectrum of CuO nanoparticle. The spectrum reveals key functional groups, with a broad peak at 3429.91 cm⁻¹ corresponding to OH stretching, a peak at 1636.87 cm⁻¹ indicating C = C stretching, and a significant peak at 516.88 cm⁻¹ confirming the Cu–O bond formation. These peaks suggest the successful formation of copper oxide nanoparticles

Spectrum of *Aspergillus niger* MN PP792979.1 filtrate, indicating key functional groups and the production of CuO nanoparticles. The total production of CuO nanoparticles was 7.4 mg/100 ml. The broad absorption peak around 3432 cm^− 1^ corresponds to O-H stretching vibrations, associated with hydroxyl groups due to absorbed water molecules on the surface of CuO. The peak at 2972.41 cm^− 1^ is attributed to C-H stretching vibrations. Distinct absorption bands at 1642.47 cm^− 1^ and 1634 cm^− 1^ correspond to C = C stretching vibrations, characteristic of water-soluble unsaturated components of the fungal extract [38–39]. The absorption band at 518 cm^− 1^ is assigned to the Cu-O bond, (Fig. 8).

### Transmission Electron Microscopy (TEM)

**Fig. 9:**
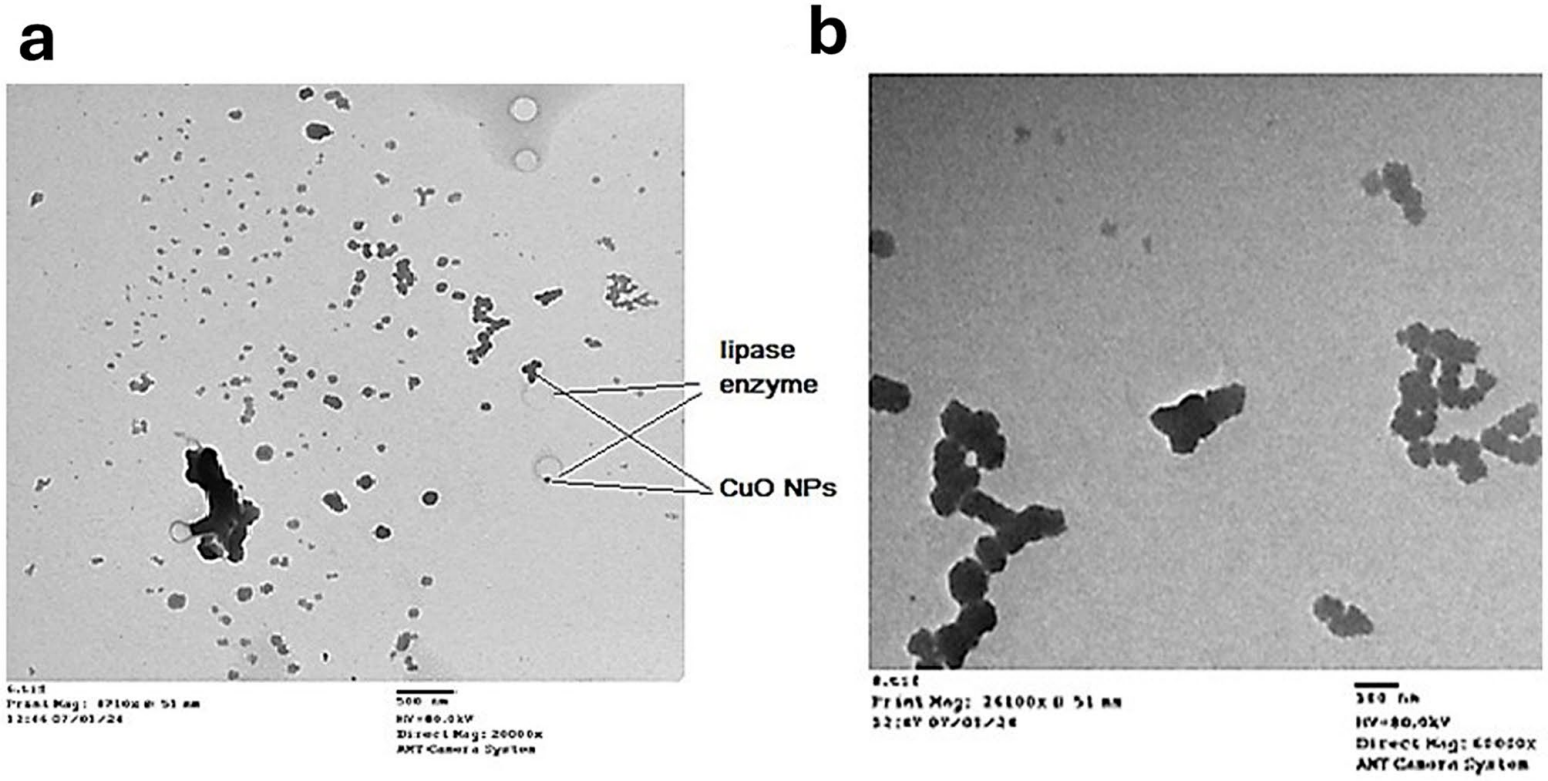
TEM of CuO nanoparticles and - protein conjugate produced by *Aspergillus niger* MN PP792979.1. (**a**) Transmission Electron Microscopy (TEM) image of CuO nanoparticles (NPs) and conjugated lipase enzyme produced by *A. niger* MN showing the linking between the lipase molecule and CuO nanoparticles that confirmed coniguate formation. (**b**) Higher magnification TEM image showing the morphology and uniform size distribution of the CuO nanoparticles which appeared as distinct well-dispersed spherical particles with average size of 71.035 nm

Analysis of *Aspergillus niger* MN PP792979.1 filtrate demonstrated the successful biosynthesis of copper oxide (CuO) nanoparticles, which appeared as distinct, well-dispersed spherical particles with average size 71.035 nm. High magnification TEM images confirmed the uniform size distribution of these nanoparticles as shown in (Fig. 9). Additionally, the TEM images indicated the presence of lipase enzyme associated with the CuO nanoparticles, suggesting a potential enzymatic role in the nanoparticle synthesis process. These findings emphasize the effective production of CuO nanoparticles by *A. niger* MN PP792979.1 and highlight their uniformity.

**Fig. 10:**
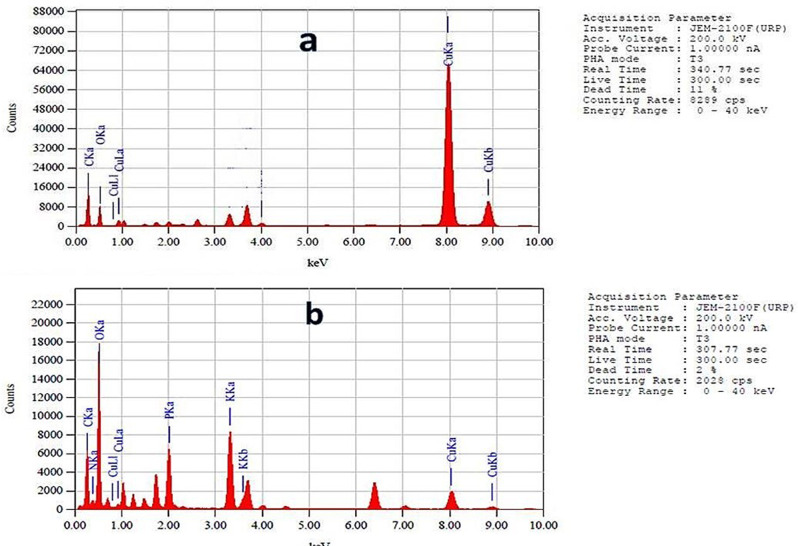
Energy Dispersive X-ray analysis (EDAX) of CuO nanoparticles and - protein conjugate by *Aspergillus niger* MN PP792979.1. (**a**) Energy Dispersive X-ray (EDAX) analysis spectrum of the biosynthesized CuO nanoparticles, displaying prominent peaks corresponding to copper (Cu) and oxygen (O), confirming their elemental composition. (**b**) EDAX analysis spectrum of CuO nanoparticles lipase conjugates

Energy Dispersive X-ray (EDAX) analysis of the biosynthesized CuO nanoparticles produced by *Aspergillus niger* MN PP792979.1 confirmed their elemental composition. The EDAX spectra displayed prominent peaks corresponding to copper (Cu), Carbon (C) and oxygen (O), validating the successful formation of CuO nanoparticles. In the first spectrum (Fig. 10a), significant Cu peak was observed at approximately 8 keV, C peak was noted around 0.2 keV and O peak was noted around 0.5 keV with mass 62.22, 30,8 and 6.22%, respectively. Similarly, the second spectrum (Fig. 10b) confirmed the structure of the produced conjugate with the presence of the lipase enzyme elements as carbon, nitrogen and phosphorus due to the used buffer with mass of 36.71% Carbon, 0.22 Nitrogen, 53.82% Oxygen, 4.79% Phosphorus, 3.97% Cu and 0.049 potassium peaks. The absence of other significant peaks indicated minimal impurities, confirming the high purity of the biosynthesized CuO nanoparticles and the conjugate.

**Fig. 11:**
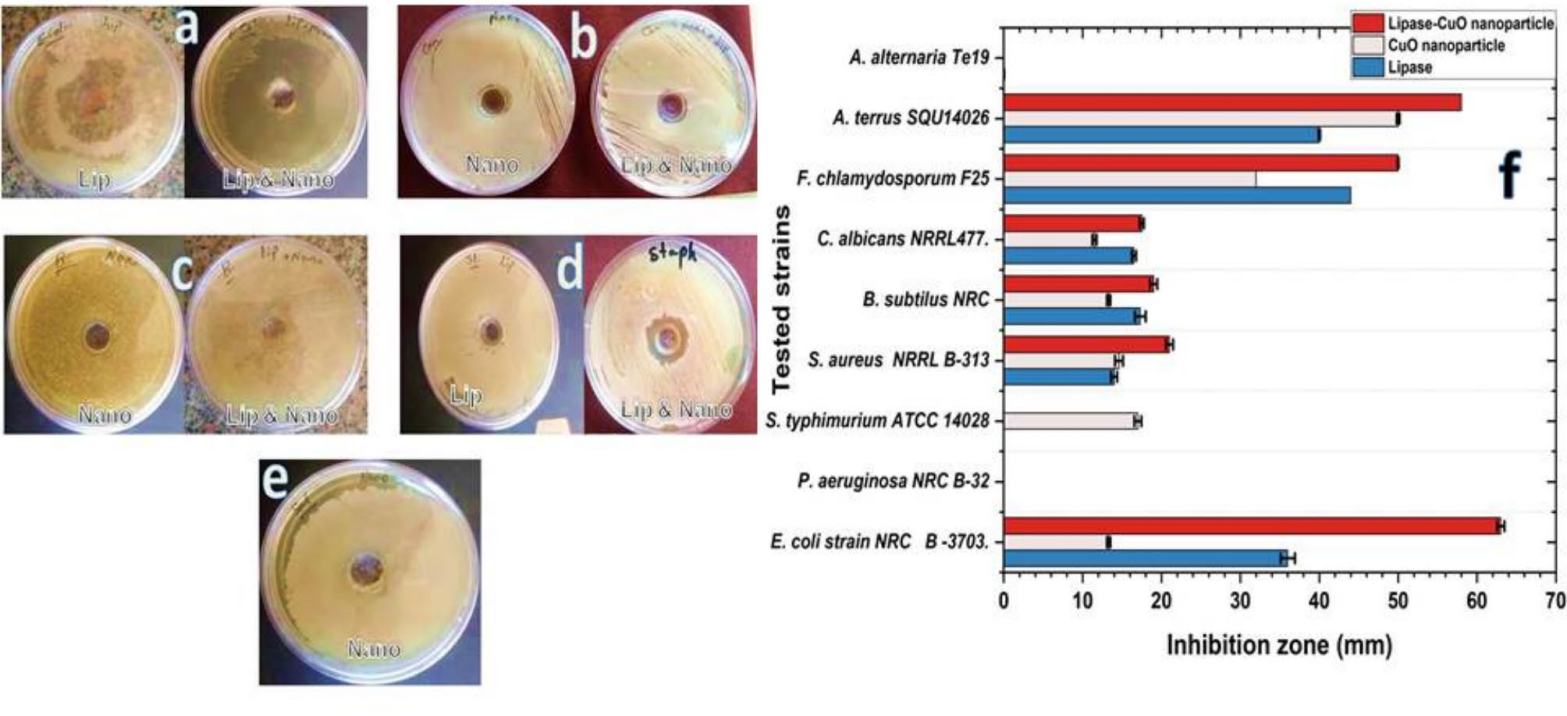
Antimicrobial Activity of Purified Lipase-CuO Conjugate. Antimicrobial activities of Lipase-CuO nanoparticle conjugate against different microbial strains. (**a**) *Escherichia coli*, (**b**) *Candida albicans* (**c**) *Bacillus subtilus*, (**d**) *Staphylococcus aureus*, (**e**) *Salmonella typhimurium*, (**f**) Histogram of CuO- lipase conjugates antimicrobial activity against tested organisms. The results indicated the synergistic effect in conjugate represented by an increase in the diameter of inhibition zone particularly against *E. coli* strain NRC B-3703 with remarkable increase of 373.6% and 75% compared to that of individual CuO nanoparticles and lipase enzyme, respectively

The antimicrobial activity of the lipase-CuO conjugate was assessed against several microbial strains (Fig. 11 and Table S5). For *Aspergillus terreus* SQU14026, the inhibition zone for the lipase-CuO conjugate was 58 mm, compared to 50 mm and 40 mm for CuO nanoparticles and lipase, respectively. In the case of *Fusarium chlamydosporum* F25, the lipase-CuO nanoparticles conjugate showed an inhibition zone of about 50 mm, with CuO nanoparticles displaying an inhibition zone of 32 mm and lipase showing 44 mm.

As for *Candida albicans* NRRL477, the lipase-CuO nanoparticles conjugate exhibited an inhibition zone of 17.5 mm, while CuO nanoparticles and purified lipase showed inhibition zones of 11.5 mm and 16.5, respectively. While, in case of *Bacillus subtilis* NRC, the inhibition zones were 19 mm for the lipase-CuO nanoparticles conjugate, 13.3 mm for CuO nanoparticles, and 17.3 mm for purified lipase. Additionally, *Staphylococcus aureus* NRRL B-313, the lipase-CuO nanoparticles conjugate showed larger inhibition zones of 21 mm compared to 14 and 14.7 CuO nanoparticles and purified lipase, respectively. Lastly, against *E. coli* strain NRC B-3703, the lipase-CuO nanoparticles conjugate demonstrated a significant inhibition zone of approximately 63 mm, while 13.3 mm for CuO nanoparticles and 36 for purified lipase. No inhibition activity was recorded against *Alternaria alternata* Te19 and *Pseudomonas aeruginosa* NRC B-32as shown in (Figure, 9). These results indicated that the lipase-CuO conjugate has enhanced antimicrobial properties compared to CuO nanoparticles and lipase individually, particularly against *E. coli* strain NRC B-3703 with remarkable increase of 373.6% and 75% followed by *S. aureus* with increase of 50 and 42.8% compared to that of individual CuO nanoparticles and lipase enzyme, respectively. All the provided results were compared to negative control with the addition of 0.1 ml DMSO instead of conjugate, while in positive controls ampicillin and mycostatin with concentrations of 50 and 100 µg/ml, respectively was used. MIC of the three tested substances (lipase only, CuO-NPs only and lipase CuO-NPs -conjugate) was determined and results indicated that the lipase-CuO conjugate has enhanced antimicrobial properties compared to CuO nanoparticles alone and lipase individually, since MIC of the conjugate was at lower concentrations than lipase and CuO nanoparticles as showed in Table S6.

## Discussion

Lipase production by fungi is a well-studied area due to the enzyme’s wide range of applications, including in the food, detergent, pharmaceuticals, and biofuel industries. Fungal lipases are highly active, making them suitable for large-scale production [40–44].

Microbial lipase production is influenced by physiological and nutritional growth parameters i.e., temperature, pH, lipid, carbon source, presence of surfactants and time. Our results indicated that castor oil is the most effective substrate for promoting microbial lipid degradation and lipase production, followed by frying oil, while engine oil is the least effective when used individually with productivity 937.6 U/ml, 787.6 U/ml and 387.6 U/ml, respectively. These results may be attributed to the structural nature of castor oil which is a vegetable oil that extracted from castor beans with about 40 to 60% seed oil content, it contains a mixture of triglycerides with about 90% of fatty acids including ricinoleic, oleic, stearic, palmitic, linoleic, linolenic acid and others that are valuable nutritional components. Also, Castor oil has low amount of saturated and polyunsaturated fatty acids that maximize its stability [45].

In case of frying oil, it resulted in near lipase productivity when used compared to castor. The results may vary according to many factors including the composition of the frying oil and the times of being used in frying, type of fatty acids liberated from the oil during frying. Three agents are responsible for changing oil composition during frying. The food water, high temperature and oxygen lead to chemical reactions in the frying oil and produce di- and monoacylglycerols, glycerol, and free fatty acids. In addition, the maximum free fatty acid content for frying oil is 0.05–0.08% [46–47]. Frying oil in this research was soybean, sunflower mixed waste frying oil has been used for frying 3 times.

Concerning the waste engine oil, the complexity of its composition led to the lowest lipase productivity by *Penicillium griseofulvum* P-1707. It is composed of oxidative oil products, impurities as chlorinated hydrocarbons and other organic compounds as benzene or naphthalene, products of decomposition of additives and metals. In addition, it becomes contaminated after a cycle of use resulted in other secondary byproducts [48].

In agreement with these recent findings, Amara and Salem [49]. reported lipases production Using *Pseudomonas aeruginosa* from castor oil waste in a mesophilic and thermophilic environment. They stated that two *P. aeruginosa* strains produced lipases with maximum activity 1121.00 U/ml and 470.82 U/ml at 37 °C & 358.72 U/ml and 147.18 U/ml at 75 °C, respectively. Also, Braga et al. [50] approved the production of lipase using castor oil by two strains of *Yarrowia lipolytica*, for *Y. lipolytica* W29 extracellular activity of lipase was 449 ± 29 (U L-1) and 516 ± 27 (U L-1) With cells centrifugation and Without cells centrifugation, respectively. While for *Y. lipolytica* IMUFRJ 50,862 the extracellular activity of lipase was 118 ± 38 UL^− 1^ and 205 ± 26 UL^− 1^ with cells centrifugation and without cells centrifugation, respectively.

In addition, Ferreira et al. [51] stated that Lipases represents the third most commercialized group of enzymes worldwide and those of microbial origin are sought for their multiple advantages. Their study aimed to produce yeast lipases from waste frying oil (WFO) by submerged fermentation (SF) from *Moesziomyces aphidis* BRT57 Yeast with maximum production of lipases activities of 8.88 and 11.39 U mL − 1, respectively.

In contrast, other studies revealed that soybean oil was the most commonly used inducer for lipase production [52–53].

Optimization of lipase production by *Penicillium griseofulvum* P-1707 was conducted using a factorial design model to evaluate the effects of five factors: temperature, initial pH, incubation time, inoculum size, and castor oil concentration. The factorial design approach enabled the identification of critical factors influencing lipase production. This insight provides a foundation for optimizing conditions to maximize lipase production in future experiments. The plackett-Burman model used to evaluate lipase production by *Penicillium griseofulvum* P-1707 identified temperature (A), castor oil (E), and interaction terms BE (pH * Castor oil) and CE (Incubation time * Castor oil) as significant model terms. It led to an increase of lipase productivity from 937.6 to 1150 U/ml. For the second design, the optimization process also resulted in a substantial increase in lipase productivity up to 2800U/ml (143.43% more than first design productivity), this may be due to that, When the three oils mixed, it resulted into higher ratio and different types of fatty acids with the effect of water presence with the enriched constituents being present in their compositions that was more preferred for microbial utilization and lipase production. The optimum conditions were at a temperature of 50 °C, an initial pH of 8, an incubation time of 5.0 days, an inoculum size of 1%, and concentrations of 1% for the tree oils (frying, engine waste and castor oils). Also, (B-Initial pH, C-Incubation time, F-engine waste oil, temperature * frying oil, Inoculum size * frying oil and frying oil * Castor oil) were found to be significant model terms.

These findings align with previous studies, such as those by Geoffry and Achur [54], Sharma et al. [55] and Kumar et al. [56], which also highlighted the importance of temperature and substrate concentration in optimizing lipase production. The novel inclusion of engine waste oil as a significant factor provides new insights into optimizing lipase production. These results underscore the effectiveness of using factorial design to identify critical variables and optimize conditions for enhanced lipase production.

Similarly, other findings reported production of lipase enzyme by four fungal isolates using oily residues (*Penicillium* sp., *Aspergillus niger*, *Aspergillus* sp., and *Aspergillus* sp.). Their results highlighted the successful optimization and purification strategies employed to enhance lipase productivity and purity. A complete factorial design 3^2^ was performed, evaluating the temperatures (28 °C, 32 °C, and 36 °C) and soybean oil inducer concentration (2%, 6%, and 10%). Each lipase-producing isolate reacted differently to the conditions tested, the *Aspergillus* sp. F18 reached maximum lipase production, compared to others, under conditions of 32 °C and 2% of oil with a yield of 11,007 µg mL^− 1^. *Penicillium* sp. F04 achieved better results at 36 °C and 6% oil, although for *Aspergillus niger* F16 was at 36 °C and 10% oil and *Aspergillus* sp. F21 at 32 °C and 2% oil [53].

Another recent study was that microbial lipases production was performed using non-sterile culture technique and hydrolysis of waste frying oils using locally isolated cold-adapted bacteria. The psychrotolerant *Pseudomonas yamanorum* was determined to have the highest lipase activity. It was found that a combination of restricted nutrient availability, low temperature and high inoculum volume prevented microbial contaminants under non-sterile conditions. The most favorable parameters for lipase production under both sterile and non-sterile conditions were 15 °C temperature, pH 8, 30 mL/L inoculum volume, 40 mL/L waste frying oil concentration, 10 mL/L Tween-80 and 72 h incubation time. The maximum lipase activities in sterile and non-sterile media were determined as 93.3 U/L and 96.8 U/L, respectively [57].

The purification process of lipase in this study showed a significant increase in specific activity through each step. Starting with a crude extract, the specific activity was 1574.18 units/mg. After ammonium sulfate precipitation, the specific activity increased to 2,228.8 units/mg, achieving a 1.42-fold purification and 64.29% recovery. Further purification using Sephadex G-200 chromatography resulted in a specific activity of 152,778 units/mg, leading to a 97.05-fold purification and 7.86% recovery units/mg. These results align well with other studies, indicating the effectiveness of the purification methods used by Gupta et al. [58]. According to Ruiz et al. [59], lipase purification from *Penicillium candidum* was applied to an octylsepharose chromatography column following ammonium sulfate precipitation. After being combined, the active samples were run through a DEAE-Sephadex column. With a low recovery of activity (0.8%), the enzyme was purified to a level of approximately 36.7- fold. The specific activities of purified lipase were roughly 14,000 (U/mg).

Another Study was performed by Ferreira et al. [60] who used Partial purification of crude enzyme by Ammonium sulfate 70% saturation to the crude enzyme and the supernatant was discarded and the precipitate was resuspended with sodium phosphate buffer (50 mM/pH 7.0) 1:3 (w/v) mentioned that the values of Specific activity, before and after crude enzymatic extract partial purification, were 0.22 (UA mg^− 1^) for crude and 1.29 (UA mg^− 1^) for Partially purified enzyme. Also, Ramani and Kennedy [61] used the same precipitation technique with ammonium sulfate and obtained a 1.43 purification factor for lipase by *Pseudomonas gessardii*, while Ulker and Karaoglu [62] achieved a 3.27 factor for lipase by *Mucorhiemalis f. cortícola*.

With a total yield of 7.4 mg/100 ml CuO nanoparticles were produced by *Aspergillus niger* MN. Characterization of the produced CuO nanoparticles using the visual observation of greenish color, UV-Vis absorption spectroscopy with strong peak at 300 nm across a range of 200–700 nm which in agreement of several studies revealed the color indication and the characteristic range of CuO nanoparticles [63–64], Fourier-transform infrared (FTIR) spectrum revealed key functional groups, The broad absorption peak around 3432 cm⁻¹ corresponds to O-H stretching vibrations, indicating the presence of hydroxyl groups due to absorbed water molecules on the CuO surface. This is consistent with findings from other studies, such as those by [38–39], which also identified O-H stretching vibrations in similar contexts.

The peak at 2972.41 cm⁻¹ is attributed to C-H stretching vibrations, a common feature in organic compounds. Distinct absorption bands at 1642.47 cm⁻¹ and 1634 cm⁻¹ correspond to C = C stretching vibrations, characteristic of water-soluble unsaturated components of the fungal extract. These findings align with previous research, which has similarly identified C = C stretching vibrations in fungal extracts [38–39].

The absorption band at 518.96 cm⁻¹ is assigned to the Cu-O bond, indicating the presence of copper complexes. This is a crucial indicator of successful nanoparticle synthesis, as confirmed by other studies that have reported similar spectral features for CuO nanoparticles produced by fungal strains [38–39]. Similarly, the TEM analysis provides compelling evidence of the successful and uniform biosynthesis of CuO nanoparticles by *A. niger* MN. These findings are consistent with previous studies, validating the effectiveness of the chosen biological system and highlighting the novel aspects of this research [38–39]. In the line of our results, the EDAX analysis provides robust evidence of the successful and pure biosynthesis of CuO nanoparticles by *A. niger* MN as significant Cu peaks were observed with mass 62.22%. Also, the EDAX analysis confirmed the structure of the CuO nanoparticles produced conjugate with mass of 36.71% Carbon, 0.22 Nitrogen, 53.82% Oxygen, 4.79% Phosphorus, 3.97% Cu and 0.049 potassium peaks which indicated minimal impurities, and high purity of the biosynthesized CuO nanoparticles and the conjugate.

These findings are consistent with previous studies, validating the use of fungal strains for nanoparticle synthesis and highlighting the advantages of biological methods over conventional chemical synthesis techniques. Further studies could explore the scalability of this method and its applicability as efficient production processes [38–39].

Previous studies have reported the antimicrobial properties of CuO nanoparticles and lipase separately. For instance, Gupta et al. [65] demonstrated the antimicrobial activity of CuO nanoparticles against various bacterial strains, with inhibition zones ranging from 10 to 30 mm. Similarly, Sharma et al. [66] reported the antimicrobial effects of purified lipase, with inhibition zones up to 20 mm. The enhanced inhibition zones observed in this study for the lipase-CuO conjugate, particularly against *A. terreus* and *E. coli*, suggest a synergistic effect that significantly boosts antimicrobial efficacy. The protein corona (PC) resulted from Cu nanoparticles lipase enzyme interaction and formation of the conjugate led to enhancement of the bio reactivity of both nanoparticles and lipase enzyme against the tested pathogenic microbial strains indicated by increasing the diameter of inhibition zone.

Several studies have been approved the new approach for preparation of protein Nano conjugates due to the magnificent enhancement in the introduced applications rather than using each of them individually [67–69]. The produced CuO nanoparticles from *Aspegillus niger* MN PP792979.1 appeared as distinct, well dispersed spherical particles with average size 71.035 nm. The uniformity, small size and spherical shape characteristics of CuO nanoparticles facilitated the interaction with lipase enzyme. The combination of lipase and CuO nanoparticles appears to enhance the antimicrobial properties beyond the sum of their individual effects. This synergistic interaction has been noted in other studies, such as those by Kumar et al. [56], who observed enhanced antimicrobial activity in conjugates of enzymes and metal nanoparticles.

In this concern, lipase – nanoparticle interactions may be produced and stabilized because of the presence of free amine groups or cysteine residues. Also, it can be initiated through the electrostatic attraction of negatively charged carboxylate groups, specifically in enzymes, particularly in enzymes that retain their biocatalytic activity in the bioconjugate material [70–71]. There are a number of ways to create an enzyme-nano association, including covalent or non-covalent attachment on modified matrices or enzyme immobilization onto nano-based support, which increases the stability and half-life of the enzymes by preventing contamination with other substances, but may also cause the nano-enzyme to interact with other existing enzymes [72–75]. The latter approach may result in a longer half-life for the used enzymes, reduced degradability during the particular processes and modifications to their kinetic and diffusion properties [76].

Using of enzymes attached to nanoparticles can help to overcome some of the problems and associated with enzyme drugs as poor stability, potential immunogenicity, and potential systemic toxicity [77]. Biomolecular conjugation is still a research topic because the ideal NP bioconjugation chemistry would enable any biological to be uniformly and reproducibly attached to any NP with control over ratio, separation distance, affinity, and display orientation. However, it is currently unable to record all of these requirements so, uncontrolled routes can be developed facing issues as extensive purification, environmental or stability constraints, random incorporation or heterogeneity resulting in mixed avidity, irreversible modifications impacting function [78–82].

Also, because of their broad range activity, ease of penetration of challenging membrane barriers, transport, long-term suppression of intracellular infections, and sterilizing capabilities, nanoparticle conjugates are regarded as alternative antibacterial and antiviral medicines. The idea of creating drug conjugates with numerous functions and appealing physiochemical characteristics in nanoparticle form would undoubtedly transform clinical medicine and be crucial in reducing the burden of disease [83].

The majority of the research showed that NP-conjugates possess antiviral, antifungal, antibacterial, antimycobacterial, anti-protozoal, and antimalarial properties. Although the mechanism of action of the majority of antimicrobial and antiviral nanoparticles is still unclear and is currently being studied systematically, studies showed that NPs could interfere with the degradation of bacterial cell membranes and the bactericidal effect of nanoparticle conjugates could be owing to reducing respiration and killing the pathogen by blocking its oxygen supply [84–86]. These claims supported our results of inhibition zone differences among tested pathogen due to the difference of their cell wall structure being gram negative or gram positive bacteria, yeast or filamentous fungi which affects the degree of penetration of the tested conjugate resulting in variant inhibition zones. Also the concentration and the diameter of the well in the tested plates considered as interfering factors [87].

Safety considerations, environmental impacts, health risks, and societal ramifications all influence whether or not nanotechnology is used. There is still a gap in our understanding of their safety and health hazards for both consumers and researchers [83], but the additional enhancement provided by the lipase in this study further supports the potential of enzyme-nanoparticle conjugates in antimicrobial applications usage after extensive investigations.

## Conclusion

In conclusion, the statistical optimization provided a rapid and effective tool for microbiological compounds production. The lipase productivity has been increased up to 143.43% (2800 U/ml). In addition, lipase- CuO nanoparticle conjugate’s antimicrobial activity shows notable improvements against several test pathogens that have potential health problems particularly *E. coli* strain NRC B-3703 with remarkable increase of 373.6% and 75% followed by *S. aureus* with increase of 50 and 42.8% compared to that of individual CuO nanoparticles and lipase enzyme, respectively. The observed synergistic effects and broad-spectrum activity highlighted the potential of this conjugate for various antimicrobial applications. These findings are consistent with previous studies, validating the effectiveness of combining enzymes with metal nanoparticles to enhance antimicrobial properties. Formation of protein-nano conjugates (protein corona) either by adsorption or chemical interaction needs to be extensively studied to ensure the stability over time range, monitoring the cytotoxicity effects on human or animal cells. Also, further research could explore the mechanisms underlying these synergistic effects and the potential for scaling up this approach for practical applications. Also, utilizing these conjugates to enhance drug delivery systems offers a vast area of study that leads to significant advancements in medical applications.

## Electronic supplementary material

Below is the link to the electronic supplementary material.

Supplementary Material 1

## Data Availability

The datasets used and/or analyzed during the current study are available from the corresponding author on reasonable request.
