# Supplementary material for: Design and assessment of lipase-CuO nanoparticle conjugates for enhanced antimicrobial efficacy against clinical pathogens
Source: BMC Biotechnol. 2025 Feb 7;25:16. doi: 10.1186/s12896-025-00950-0 (PMC11806700; doi:10.1186/s12896-025-00950-0)
Supplement: Supplementary file 1 — Supplementary Material 1 [file 12896_2025_950_MOESM1_ESM.docx]

**Table S1. Experimental Runs:** **Plackett-Burman Design:** The design matrix for the first model is as follows:

| **Run** | **A: Temperature (°C)** | **B: Initial pH** | **C: Incubation Time (Days)** | **D: Inoculum Size (%)** | **E: Castor Oil (%)** |
| --- | --- | --- | --- | --- | --- |
| 1 | 37.5 | 6 | 5 | 5.5 | 3 |
| 2 | 25 | 4 | 2 | 1 | 1 |
| 3 | 37.5 | 6 | 5 | 5.5 | 3 |
| 4 | 50 | 4 | 8 | 10 | 1 |
| 5 | 25 | 4 | 8 | 1 | 1 |
| 6 | 25 | 8 | 8 | 10 | 1 |
| 7 | 50 | 8 | 8 | 1 | 5 |
| 8 | 25 | 4 | 2 | 10 | 5 |
| 9 | 25 | 8 | 2 | 10 | 5 |
| 10 | 50 | 8 | 2 | 10 | 1 |
| 11 | 37.5 | 6 | 5 | 5.5 | 3 |
| 12 | 25 | 8 | 8 | 1 | 5 |
| 13 | 50 | 8 | 2 | 1 | 1 |
| 14 | 50 | 4 | 8 | 10 | 5 |
| 15 | 50 | 4 | 2 | 1 | 5 |

*Each run represents a unique combination of factors, allowing for a comprehensive analysis of their impact on lipase production.

**Table S2. Experimental Runs for the Second Design Model.**

| **Run** | **A: Temperature (°C)** | **B: Initial pH** | **C: Incubation Time (Days)** | **D: Inoculum Size (%)** | **E: Frying Oil (%)** | **F: Engine Waste Oil (%)** | **G: Castor Oil (%)** |
| --- | --- | --- | --- | --- | --- | --- | --- |
| 1 | 35 | 6 | 5 | 5.5 | 3 | 3 | 3 |
| 2 | 20 | 4 | 2 | 1 | 1 | 1 | 1 |
| 3 | 35 | 6 | 5 | 5.5 | 3 | 3 | 3 |
| 4 | 50 | 4 | 8 | 10 | 5 | 1 | 1 |
| 5 | 20 | 4 | 8 | 1 | 5 | 5 | 1 |
| 6 | 20 | 8 | 8 | 10 | 1 | 1 | 1 |
| 7 | 50 | 8 | 8 | 1 | 1 | 1 | 5 |
| 8 | 20 | 4 | 2 | 10 | 1 | 5 | 5 |
| 9 | 20 | 8 | 2 | 10 | 5 | 1 | 5 |
| 10 | 50 | 8 | 2 | 10 | 5 | 5 | 1 |
| 11 | 35 | 6 | 5 | 5.5 | 3 | 3 | 3 |
| 12 | 20 | 8 | 8 | 1 | 5 | 5 | 5 |
| 13 | 50 | 8 | 2 | 1 | 1 | 5 | 1 |
| 14 | 50 | 4 | 8 | 10 | 1 | 5 | 5 |
| 15 | 50 | 4 | 2 | 1 | 5 | 1 | 5 |

*Each run represents a unique combination of the seven factors, enabling a comprehensive analysis of their impact on lipase activity.

**Table S3: ANOVA for First Design Model**

| **Source** | **Sum of Squares** | **df** | **Mean Square** | **F-value** | **p-value** |  |
| --- | --- | --- | --- | --- | --- | --- |
| **Model** | 4.933E+05 | 9 | 54814.81 | 6.58 | 0.0427 | significant |
| A-temperature | 1.344E+05 | 1 | 1.344E+05 | 16.13 | 0.0159 |  |
| B-Initial pH | 27777.78 | 1 | 27777.78 | 3.33 | 0.1419 |  |
| C-Incubation time | 1111.11 | 1 | 1111.11 | 0.1333 | 0.7335 |  |
| D-Inoculum size | 1111.11 | 1 | 1111.11 | 0.1333 | 0.7335 |  |
| E-Castor oil | 83333.33 | 1 | 83333.33 | 10.00 | 0.0341 |  |
| AE | 54444.44 | 1 | 54444.44 | 6.53 | 0.0629 |  |
| BE | 1.878E+05 | 1 | 1.878E+05 | 22.53 | 0.0090 |  |
| CE | 1.344E+05 | 1 | 1.344E+05 | 16.13 | 0.0159 |  |
| DE | 27777.78 | 1 | 27777.78 | 3.33 | 0.1419 |  |
| Curvature | 10666.67 | 1 | 10666.67 | 1.28 | 0.3211 |  |
| **Residual** | 33333.33 | 4 | 8333.33 |  |  |  |
| Lack of Fit | 6666.67 | 2 | 3333.33 | 0.2500 | 0.8000 | not significant |
| Pure Error | 26666.67 | 2 | 13333.33 |  |  |  |
| **Cor Total** | 5.373E+05 | 14 |  |  |  |  |

**Table S4: ANOVA for Second Design Model**

| **Source** | **Sum of Squares** | **df** | **Mean Square** | **F-value** | **p-value** |  |
| --- | --- | --- | --- | --- | --- | --- |
| **Model** | 2.727E+06 | 10 | 2.727E+05 | 15.22 | 0.0231 | significant |
| A-temperature | 27613.64 | 1 | 27613.64 | 1.54 | 0.3027 |  |
| B-Initial pH | 8.438E+05 | 1 | 8.438E+05 | 47.09 | 0.0063 |  |
| C-Incubation time | 5.834E+05 | 1 | 5.834E+05 | 32.56 | 0.0107 |  |
| D-Inoculum size | 1.013E+05 | 1 | 1.013E+05 | 5.65 | 0.0979 |  |
| E-frying oil | 1875.00 | 1 | 1875.00 | 0.1047 | 0.7676 |  |
| F-engine waste oil | 1.934E+05 | 1 | 1.934E+05 | 10.79 | 0.0462 |  |
| G-Castor oil | 4934.21 | 1 | 4934.21 | 0.2754 | 0.6360 |  |
| AE | 3.901E+05 | 1 | 3.901E+05 | 21.78 | 0.0186 |  |
| DE | 5.704E+05 | 1 | 5.704E+05 | 31.84 | 0.0110 |  |
| EG | 5.868E+05 | 1 | 5.868E+05 | 32.75 | 0.0106 |  |
| Curvature | 63375.00 | 1 | 63375.00 | 3.54 | 0.1566 |  |
| **Residual** | 53750.00 | 3 | 17916.67 |  |  |  |
| Lack of Fit | 33750.00 | 1 | 33750.00 | 3.38 | 0.2076 | not significant |
| Pure Error | 20000.00 | 2 | 10000.00 |  |  |  |
| **Cor Total** | 2.844E+06 | 14 |  |  |  |  |

**Table S5: Antimicrobial Activity of CuO nanoparticles, lipase and their Conjugates Against Test Organisms**

| **Tested strains** | | **Mean zone of growth inhibition (mm)** | | | Amp | Myco |
| --- | --- | --- | --- | --- | --- | --- |
|  |  | **lipase** | **Nano** | **Lipase +Nano** |  |  |
| ***Bacteria*** | ***E. coli* strain NRC B -3703.** | 36± 0.9 | 13.3± 0.23 | 63±0.46 | 22± | n.d |
|  | ***Pseudomonas aeruginosa* NRC B-32** | **-ve** | **-ve** | **-ve** | **14** | n.d |
|  | ***Salmonella typhimurium* ATCC 14028** | **-ve** | 17 ± 0.46 | **-ve** | **15** | n.d |
|  | ***Staphylococcus aureus* NRRL B-313** | 14 ± 0.4 | 14.7 ± 0.51 | 21 ± 0.46 | 13 | n.d |
|  | ***Bacillus subtilus* NRC** | 17.3 ± 0.69 | 13.3 ± 0.23 | 19 ± 0.46 | 14 | n.d |
| **Yeast** | **Candida albicans NRRL477.** | 16.5 ± 0.28 | 11.5 ± 0.28 | 17.5 ±0.28 | n.d | 14 |
| **Fungi** | ***Fusarium chlamydosporum* F25** | 44 ±0.0 | 32 ±0.0 | 50 ±0.05 | n.d | 16 |
|  | ***Aspergillus terrus* SQU14026** | 40 ± 0.1 | 50 ±0.2 | 58 ±0.0 | n.d | 18 |
|  | ***Alternaria alternata* Te19** | **-ve** | **-ve** | **-ve** | n.d | **14** |
| ***DMSO 5%*** | | **-ve** | **-ve** | **-ve** | **-----** | **-------** |

Inhibition Zones = well diameter + growth inhibition zone; well diameter = 6 mm. The mean zone of inhibition was determined from three replicate readings; Amp= Ampicillin (bacterial positive control); Myco = Mycostatin (fungal positive control) , n. d=not determined; DMSO 5% Negative control.

**Table 6S: MIC and MBC determination**

| **Bacterial strains** | **Concentrations µg/ml** | | | | | | |
| --- | --- | --- | --- | --- | --- | --- | --- |
|  | **lipase** | | **CuO NPs** | | **Lipase +CuO NP conjugate** | | |
|  | **MIC** | **MBC** | **MIC** | **MBC** | **MIC** | **MBC** | |
| ***E. coli* strain NRC B -3703.** | 425 | 425 | 200 | 400 | 106.26 µg/ml +100 µg/ml | 212.5 µg/ml+200 µg/ml | |
| ***Salmonella typhimurium* ATCC 14028** | -- | -- | 400 | 800 | -- | -- | |
| ***Staphylococcus aureus* NRRL B-313** | 425 | 850 | 400 | 800 | 212.5 µg/ml+200 µg/ml | 425 µg/ml+400 µg/ml | |
| ***Bacillus subtilus* NRC** | 212.5 | 425 | 200 | 400 | 106.26 µg/ml +100 µg/ml | 212.5 µg/ml+200 µg/ml | |
| ***Fungal isolates*** | **MIC Concentrations µg/ml** | | | | | | |
|  | **lipase** | | | | **CuO NPs** | | **Lipase +CuO NP conjugate** |
| **Candida albicans NRRL477.** | 425 | | | | 800 | 425 µg/ml+400 µg/ml | |
| ***Fusarium chlamydosporum* F25** | 212.5 | | | | 200 | 106.26 µg/ml +100 µg/ml | |
| ***Aspergillus terrus* SQU14026** | 425 | | | | 400 | 212.5 µg/ml+200 µg/ml | |

**-Lipase concentrations (1700, 850, 425, 212.5, 106.25 and 53.13 µg/ml)**

**-CuO NPs concentrations (1600, 800, 400, 200, 100, 50, 25 and 12.5 µg/ml)**
